# Analysis of Trace Elements in Human Brain: Its Aim, Methods, and Concentration Levels

**DOI:** 10.3389/fchem.2019.00115

**Published:** 2019-03-05

**Authors:** Cezary Grochowski, Eliza Blicharska, Paweł Krukow, Kamil Jonak, Marcin Maciejewski, Dariusz Szczepanek, Katarzyna Jonak, Jolanta Flieger, Ryszard Maciejewski

**Affiliations:** ^1^Department of Anatomy, Medical University of Lublin, Lublin, Poland; ^2^Department of Neurosurgery and Pediatric Neurosurgery, Medical University of Lublin, Lublin, Poland; ^3^Department of Analytical Chemistry, Medical University of Lublin, Lublin, Poland; ^4^Department of Clinical Neuropsychiatry, Medical University of Lublin, Lublin, Poland; ^5^Department of Psychiatry, Psychotherapy and Early Intervention, Medical University of Lublin, Lublin, Poland; ^6^Department of Biomedical Engineering, Lublin University of Technology, Lublin, Poland; ^7^Institute of Electronics and Information Technology, Lublin University of Technology, Lublin, Poland; ^8^Department of Foreign Languages, Medical University of Lublin, Lublin, Poland

**Keywords:** trace element distribution, metal concentrations, post mortem material, brain tissue, spectroscopy

## Abstract

Trace elements play a crucial role in many biochemical processes, mainly as components of vitamins and enzymes. Although small amounts of metal ions have protective properties, excess metal levels result in oxidative injury, which is why metal ion homeostasis is crucial for the proper functioning of the brain. The changes of their level in the brain have been proven to be a risk factor for Alzheimer's, Parkinson's, and Huntington's diseases, as well as amyotrophic lateral sclerosis. Therefore, it is currently an important application of various analytical methods. This review covers the most important of them: inductively coupled ground mass spectrometry (ICP-MS), flame-induced atomic absorption spectrometry (FAAS), electrothermal atomic absorption spectrometry (GFAAS), optical emission spectrometry with excitation in inductively coupled plasma (ICP-OES), X-ray fluorescence spectrometry (XRF), and neutron activation analysis (NAA). Additionally, we present a summary of concentration values found by different research groups.

## Introduction

Trace elements play a crucial role in many biochemical and physiological processes in humans, being mainly components of various vitamins and enzymes (Zecca et al., 2004; Bartzokis et al., 2007). Their balance within the brain is regulated in a complex manner by brain barrier systems such as the blood-brain barrier (BBB), choroidal blood-cerebrospinal fluid barrier, blood-cerebrospinal fluid (CSF) barrier, and even CSF-brain barrier (Strazielle and Ghersi-Egea, 2013). The homeostasis of trace elements relies on the processes of absorption, distribution, biotransformation, and excretion (Zheng and Monnot, 2012). Trace ions such as Fe, Cu, Ca, Co, Mg, Mn, and Mo are essential for the proper functioning and growth of the brain as they provide protection against diseases and reactive oxygen species as second messengers, gene expression regulation, catalysis and enzyme activation (Lee et al., 2008).

On the other hand, excess amounts of elements may induce cellular damage resulting in a variety of syndromes caused by abnormal proteins, lipid peroxidation and oxidation of ROS-scavenging enzymes. Neurodegenerative diseases such as Alzheimer's disease—AD, Parkinson's disease—PD, and Wilson's disease are known to be correlated with metal content shifts within different areas of the brain (Hutchinson et al., 2005; Corbin et al., 2008; Squitti, 2012; Tiiman et al., 2013; Sharma et al., 2017) or with a disturbed distribution of these elements. For instance, a 339% increase in Zn, 466% increase in Cu and 177% increase in Fe, and a 4,653% increase in Ca was found in human plaque of AD patients in comparison to healthy subjects (Leskovjan et al., 2009). As another example, iron, which is highly concentrated in neuromelanin is suspected of being a cellular susceptibility factor in Parkinson's disease, as well (Depboylu et al., 2007).

Therefore, the analysis of metal content in the brain material is a very interesting application of trace metal analysis. Besides the disease diagnostics, it can also be used to trace the action of drugs, including metal chelators, which are believed to be one of current treatment ideas (Tõugu et al., 2009).

The number of papers presenting this approach has been growing significantly in the last years. The basic methods of trace analysis in the brain include inductively coupled ground mass spectrometry (ICP-MS), flame-induced atomic absorption spectrometry (FAAS), electrothermal atomic absorption spectrometry (GFAAS), optical emission spectrometry with excitation in inductively coupled plasma (ICP-OES), X-ray fluorescence spectrometry (XRF), and neutron activation analysis (NAA) (Brown and Milton, 2005).

The examples cited in the following part of the review show that the content of elements in human brain samples can be examined by many methods. The type of method used depends on the type of information we want to obtain (quantitative analysis, qualitative analysis, speciation analysis, distribution of the analyte in the sample). The choice of the appropriate analytical method is also dependent on the parameters of the method, such as the limit of detection and determination, accuracy and precision, sensitivity, and selectivity (Van Loon and Barefoot, 1992).

There is no easy way to recommend a method for the particular task. Every method summarized in this article can determine trace metals in a similar range of concentrations and does not differ substantially in detection limits and expenses (Brown and Milton, 2005). Therefore, the minor factors can be a crucial thing for a decision. ICP-MS and ICP-OES seems to be most universal and frequently used. Table 1 presents a list of analytical methods used for qualitative and quantitative analysis of elements in human brain samples.

**Table 1:** The representative examples of the application of various methods in metal analysis in brain samples.

**Method**	**Determined elements**	**References**
FAAS	Ca, Cu, Fe, Mn	Goldberg and Allen, 1981
FAES	K, Na,	Ramos et al., 2016
GFAAS (ETAAS)	Al, Fe, Sr, Se,	Xu et al., 1992; Ejima et al., 1996; Peltz-Császma et al., 2005; Gała̧ zka-Friedman et al., 2011
TH GFAAS	Al,	Mirza et al., 2017; Mold et al., 2018
ICP-MS	Ag, As, Ba, Ca, Ce, Cr, Cu, Fe, K, Li, Mg, Mn, Na, Ni, Se, Pb, Rb, Sn, Sr, Ti, V, Y, Zn	Rembach et al., 2013; Krebs et al., 2014; Dahlberg et al., 2015; Ramos et al., 2015, 2016; Korvela et al., 2016
LA-ICP-MS	Al, C, Cu, F, Fe, P, Pb, S, Si, U, Th, Zn,	Becker et al., 2004, 2005, 2007a; Zoriy and Becker, 2007; Becker and Salber, 2010
MC ICP-MS	S,	Yu et al., 2017
ICP-OES	Al, As, Ca, Cd, Cu, Cr, Fe, K, Mg, Mn,Na, Ni, P, Pb, S, Si, Sr,Zn,	Andrasi et al., 1995; Andrási et al., 1999; Rajan et al., 1997; Faa et al., 2001; Peltz-Császma et al., 2005; Korvela et al., 2016
XRF	Ca, Cl, Cu, Fe, K, P, S, Zn,	Wandzilak et al., 2015
SR XRF	Ca, Cl, Cu, Fe, K, Mn, Ni, P, S, Zn,	Sartore et al., 2017
PIXE	Ca, Cu, Fe, K, Mn, P, Se, Rb, Zn,	Duflou et al., 1989; Hebbrecht et al., 1999; Stüber et al., 2014
NAA	Ce, Cs, Dy, Eu, Gd, K, La, Lu, Na, Nd, Rb, Sm, Tb, Yb,	Koeberl and Bayer, 1992; Bélavári et al., 2004
INAA	Ag, Cs, Ba, Br, Fe, Eu, K, Na, Se, Rb, Zn,	Part, 2001; Leite Jacob-Filho et al., 2008

### Sampling Issues

The brain tissue is a complex matrix. In the human brain, the total fat content is about 30% (on a dry matter basis; Suzuki and Suzuki, 1972), and the water content is around 70–80% (Császma et al., 2003). The dry part is built mainly from lipids: about 40% of cholesterol, about 15% of glycolipids, about 15% phospholipids, and about 5% of sphingomyelin. The rest of dry matter contains mainly proteins (Gonzalez-Riano et al., 2016).

The brain tissues analyzed for the metal contents are mainly collected *post mortem* (from autopsy), as brain biopsy procedures are performed only for analysis of tumors for cancer diagnostics. It is generally recommended to freeze the samples deeply (in liquid nitrogen) to avoid any metabolism. Although the total metal content does not change during the chemical processes, the redox reactions can significantly alter the results if speciation analysis is required.

It can be assumed that an analyst is not directly responsible for proper sampling of the tissues from the brain, as the sampling is done mainly by qualified forensic physicians during autopsy at diagnosis of the disease (Hynd et al., 2003). However, the circumstances of the death (coma, hypoxia, hyperpyrexia at the time of death etc.) and the time period between death and autopsy can significantly change brain composition (Stan et al., 2006). A minimal set of samples for histopathologic examination consists currently of 12 brain fragments: middle frontal gyrus, cingulate gyrus, superior and middle temporal gyri, hippocampus and parahippocampal gyrus, inferior parietal lobule, putamen and globus pallidus, midbrain, pons, caudate nucleus; cerebellar vermis hemisphere (including the dentate nucleus), and medulla (Love, 2004). The hippocampus and cerebellum are considered to be two most important parts of the brain in the diagnostic context (Gonzalez-Riano et al., 2016).

In general, the tissues are deeply frozen after autopsy collection. For most of the methods they have to be mineralized, mainly by acid digestion, or leave solid, so there is no risk of extraction losses (Bodzon-Kulakowska et al., 2007; Xue et al., 2012). However, the analyst must be aware of other loss possibilities. Each sampling step should be based on careful rinsing. Moreover, surfaces of improper containers can absorb trace elements. Trace elements can also be volatile so each sampling step should be performed in closed containers. This does not apply to laser ablation where sampling is done from an unprocessed tissue. Nevertheless, the more significant problem (than the losses) is the risk of contamination. It is particularly important to use a dedicated grade reagents and solvents and sample storage containers made with metal-free materials.

### Quality Assurance Issues

Regardless of contamination and losses, the quality of the analysis can be affected by many other factors (Parr, 1985). It is generally assumed that only certified reference materials (CRM, or at least standard reference materials, SRM) can prove the quality of a method in a particular laboratory (Parr, 1985). These materials are reference samples that have to be analyzed with the same method to prove that the results do not differ significantly. During the choice of the materials, the maximum similarity to brain tissue (in the context of chemical composition) should be achieved as there is no reference brain tissue available on the market (Gallorini, 1995; Gallorini and Apostoli, 1996). The examples used in the research are: bovine liver SRM 1577b, bovine muscle powder SRM 8414, whole egg powder SRM 8415, and oyster tissue SRM 1566b (Leite Jacob-Filho et al., 2008; Batista et al., 2009).

## Spectroscopic Methods

The choice of an analytical method should be dependent on the purpose of the analysis and the limit of determination of a given method. Additionally, the type of research material is equally relevant. If the test is to determine the qualitative and quantitative content of the elements, it is necessary to opt for spectroscopic methods. However, if it is also important to know the spatial distribution of analytes on the surface of the tissue and to determine the state of elements occurrence, the method of surface imaging of the sample should be applied by mapping the elements quantitatively, e.g., laser ablation with detection in a mass spectrometer with ionization in inductively coupled plasma, LA ICP-MS (laser ablation inductively coupled plasma mass spectrometry). The results of the analyses should be based on a proper sample preparation procedure and on an analysis using validated methods ensuring traceability of the test sample results.

### ICP-MS

One of the most popular methods of analysis of content of elements in brain tissue samples is the inductively coupled plasma mass spectrometry—ICP-MS. This method is based on the generation of single-positive ions of the determined elements in the strictly parameterized plasma (the amount of ions with a double charge is strictly limited and usually should not exceed 3% of all charged particles). These ions, after passing through properly constructed ion optics (aimed at separating the photonic background), are identified on the basis of the mass/charge ratio (m/z) using a mass separator and a detector. Different types of separators are used, depending on the assumed degree of ion separation. The most commonly used quadrupole separator is found in many configurations. Photomultipliers are used as detectors, which are adapted for the detection of ions by placing on their optical path scintillation crystals that convert the ion flux into photons. The generation of single charge ions requires precise setting of plasma parameters. The most important are temperature, electrostatic potentials (deflecting the ion beam), plasma flow and gas flow of the nebulizer. Sample depth and sampling time constitute significant parameters as well.

Due to low background signal and large number of ions produced, it is possible to obtain a very low detection limit for most elements (in the per-billion range; He et al., 2017). The most important advantages of the ICP-MS method include: high sensitivity and precision, low limits of quantitation (at μg/L, ng/L levels), exceptionally high linearity of the calibration curve including up to 9 orders of magnitude, multi-element analysis of most elements of the periodic table, relatively short time of analysis, and a small amount of sample needed to make the determination.

Physical or spectral interference is an important factor affecting the quality of results obtained by the ICP-MS method. Physical interference results mainly from the difference in viscosity and surface tension of the sample in relation to the standards used. One of the ways to eliminate this type of interference is the use of an internal standard suited to the analytes tested in terms of ionization and mass energy. In the work (Dahlberg et al., 2015) testing the content of K, Na, Mg, Ca, Zn, Fe, Cu, Mn, and Cr, the internal standard Sc, Rh, In, and Lu was used in each sample. In order to avoid interference due to mass differences, an internal standard that has a mass number as close to the analyzed element as possible should be used (however, it is not always possible to use an appropriate, stable isotope). Another method may be the use of surfactants to reduce the surface tension, e.g., ammonium salts, Tween80 or Triton X-100.

The human brain's biological matrix (containing a high concentration of organic and inorganic substances) can cause clogging of the nebulizer and deposition of the matrix on the plasma torch and cones. The solution here is the dilution of the sample and the use of specialized nebulizers (Parsons and Barbosa, 2007). It should be noted that extraction of analytes from the sample with appropriately selected extraction reagents, e.g., methyl isobutyl ketone (MIBK), may also be a good solution. However, this requires re-extraction into aqueous solutions or changing plasma parameters and preparing a series of standards in the reagent used.

Spectral interference is the effect of signal overlap from other ions (formed in certain amounts of charged double ions, or from the combination of atoms derived from plasma gas, air, water, sample matrix, or acids used to mineralize samples) on the analyte signal (Lum and Sze-Yin Leung, 2016). There are many methods to eliminate them within the method. One of solutions in sulfur and phosphorus speciation (Hinrichs et al., 2007) is the application of the combined HPLC-ICP-SFMS method (high performance liquid chromatography/high resolution mass spectrometry with inductively coupled plasma exclusion and sector mass separator). These elements are separated by means of a liquid chromatograph and then analyzed using a mass spectrometer. This solution allows to eliminate the interference of polyatoms (15N16O +, 14N16O1H +, 14N17O +, 12C18O1H + in the case of 31P and 16O16O +, 15N16O1H +, 14N18O + for the 32S1H216O2, and 16O18O + isotope) and thus increasing the selectivity. Another solution is to use the collision reaction (CRI) chamber. Ions such as 40Ar16O are broken down inside by a gas supplied with a constant, strictly defined stream, e.g., hydrogen. The reaction generates atomic argon, hydrogen ions, and water.

In recent years, the use of combined analytical methods using chromatographic methods with ICP- MS (gas chromatography (GC), high performance liquid chromatography (HPLC), size exclusion chromatography (SEC), ion chromatography (IEC), reversed phase chromatography (RPLC), ion pair chromatography IPC), and for the assays the aforementioned spectroscopic methods (FAAS, GFAAS, ICP-OES, ICP-MS) has become very popular. For instance (Wolf et al., 2003), we will find a combination of the ICP-MS method with CZE (capillary electrophoresis), which was used to study the content of metallothioneins (low-molecular proteins involved in the detoxification of organisms from harmful metal ions) in the human brain. This combined method makes it possible to obtain a high resolution, perform multi-element analysis of small sample volumes at very low limits of determination.

ICP-MS methods have been recently very popular when combined with one of the most modern sampling methods—laser ablation (LA). This technique consists in the interaction of electromagnetic radiation of the laser causing a series of physicochemical processes leading to the creation of a system consisting of a carrier gas (usually argon) and particles of test material dispersed in it (Pozebon et al., 2014, 2017). It is a laser evaporation and atomization of the sample, so that the analysis can be subjected to solid samples, without the mineralization stage.

Becker et al. (2004) studied the content of elements such as P, S, Si, Al, Cu, Zn in proteins originating from the human brain after they were separated by means of gel electrophoresis. The tests applied the ICP-SFMS method (mass spectrometry with excitation in inductively coupled plasma and sector mass separator) with micro-sampling using the LA method. In phosphorus determinations in this type of matrix, attention should be paid to high-intensity isobaric interference from individuals such as 15N16O +, 14N16O1H +, and 14N17O1. To prevent this, the measurements were carried out at a resolution of 4,000. In the case of sulfur analysis the occurrence of 16O2 ion is one of the most common disturbances.

The research carried out by Becker et al. (2003) proved a significantly lower limit of quantification for P determinations in the case of the ICP-SFMS method (20 pg/g) compared with the ICP-MS method using quadrupole and collision chamber (1.3 ng/g). The biggest advantages of using the LA method is the lack of sample destruction, easy sample preparation, and the ability to analyze transparent and opaque samples (Durrant, 1999).

### ICP-OES

Emission spectrometry with excitation in inductively coupled plasma (ICP-OES, but ICP- AES abbreviation is also used) differs from ICP-MS spectrometry by the type of detection. In this case, the emission spectrum of analytes is analyzed. The entire process of obtaining a properly parameterized plasma is carried out on the same basis. A properly prepared sample contained in the solution is delivered by means of a peristaltic pump to a plasma torch powered by plasma gas. It is most often argon, due to its relatively low price and chemical passivity, but any gas can be used for this purpose. In the past there were experiments with nitrogen and even with oxygen carried out. Inside the plasma torch, the supplied argon produces a plasma with a temperature of up to 10,000 K under the influence of the radio frequency electromagnetic field generated by the surrounding torch, where the sample undergoes drying, decomposition, atomization, and finally ionization. The conditions are chosen so that they will produce predominantly one-positive ions. Unlike in the case of mass spectrometry, the emission spectrum produced by the excited ions is subjected to analysis. The individual elements that make up the analyte emit a linear spectrum with specific wavelengths corresponding to their energy levels. It should be emphasized that the formed spectral lines belong to ions, and not (as in atomic absorption spectrometry) to atoms. The spectrum emitted by the ions goes next to the monochromator, where one particular line of the analyzed element is separated and then measured using a radiation detector, usually a photomultiplier. The measure of the element's content in the sample is obtained from the intensity of the measured spectral line. As in the case of ICP-MS, the possibility of forming multielement ions characterized by their own molecular spectra may raise the analytical background in the case of some analyses.

The ICP-OES analysis is characterized by the limit of detection at the level of mg/L and μg/L, and in some cases even ng/L. This puts it more or less between the ICP-MS and FAAS methods. Nevertheless, there are areas of application where it is particularly useful. They are, among others, sulfur and phosphorus analyses, with which the ICP-MS method is not particularly successful, while FAAS also does not allow to reach the expected low levels. The sensitivity and precision of the ICP-OES method due to the use of plasma excitation is similar to other techniques of this type. The high linearity of the method is also very characteristic.

Korvela et al. (2016) conducted multielementic analysis of cerebrospinal fluid samples using the ICP-MS method (47 Ti, 51 V, 55 Mn, 61 Ni, 66 Zn, 75 As, 85 Rb, 88 Sr, 107 Ag, 118 Sn, 138 Ba, and 208 Pb isotopes tested) and ICP-AES (Ca, Cu, Fe, Mg, P, S, Si, Sr, Zn, K, and Na tested). In the case of Sr, As, Ba, Ti, Rb, Ca, Mg, P, K, and Na, sufficient high signals were found (concentration was above the limit of quantification) and RSD (relative standard deviation) with values below 10%.

### FAAS

The FAAS method is one of the simplest and fastest analytical methods for trace elements determination. Its principle is based on one of Kirchoff's spectroscopic laws. According to it, the cooler gas, surrounding hot emission source, eliminates spectral lines from the spectrum of the source corresponding to its specific energy levels. On this basis, many analytical apparatuses have been constructed that differ in the way in which this absorbing gas is obtained, or the atomization method. One of the techniques is atomization in the FAAS flame. The light from a specially built lamp, called a hollow cathode lamp (HCL), is passed through a long, narrow flame playing the role of an atomizer, where the processes of light absorption at a specific wavelength occur, corresponding to the energy levels of the sample introduced into the flame. Then the weakened light of this wavelength is separated from the rest of the radiation spectrum by means of a monochromator (Czerny-Turner one is most often used) and directed to the measurement with a photomultiplier. Only the general principle of operation is described above. There are many modifications—starting with the emission source: EDL electrode less lamps, where the emission occurs in the resonator coil, UV, and VIS full-spectrum arc lamps, through various atomization methods and various types of monochromators of light, to various detection methods.

As the atomization takes place in a flame in relatively high temperature, a significant obstacle to the use of the FAAS method is the occurrence of so-called cyan bands. This name includes high- temperature connections CN, NH, and CH causing the formation of high masking background for the elements determined. This is particularly important when using an acetylene/nitrous oxide flame characterized by high self-emission. This may, in some cases, even make analysis impossible.

### GFAAS (ETAAS)

Another way of sample atomization is to use a graphite furnace. In this case, the cloud of the atomized sample arises inside the tube of pizolithic graphite placed in the center of the specially constructed furnace and the analyzing light stream passes through it. The tube is heated to a high temperature with high current. As a result the sample is dried, decomposed, incinerated and atomized. The temperature in the furnace can reach a value of even more than 3,500 K. Such a high temperature, significantly exceeding even the flame temperature of acetylene/nitrous oxide mixture results in a significant reduction of the detection limit compared to the FAAS method.

In order to ensure durability of the tube and to reduce the degree of its consumption in the furnace, an anaerobic atmosphere is used, and the most commonly used gas in this case is argon.

The method is not free from an interference. An important problem is the scattering of light on the smoke particles created from the pyrolysis of organic parts, as well as the pyrolysis of the tube material itself, i.e., non-specific absorption. This results in incorrectly elevated results. Due to the high temperature of the furnace, there may also be further induction of atoms until the occurrence of an undesired ionization process. To avoid this, additives of deionizing substances are used.

It is very important to reduce the background effect on the intensity of the spectral lines of the element being measured. This is achieved by using the Zeeman effect of splitting a single spectral line into three or more components using a magnetic field. Measurements of light beams differently polarized to the magnetic field corresponding to the split lines are performed.

In addition to the limit of determination in the order of μg/L, all other validation parameters are at the level of methods including the atomic absorption spectrometry technique.

The GFAAS method has been used for the analysis of elements in the human brain for many years. The most popular elements determined in this matrix using the above mentioned method are definitely Se, Al, Fe (Xu et al., 1992; Gała̧ zka-Friedman et al., 2011). Recently, the TH-GFAAS (transversely-heated graphite furnace atomic absorption spectrometry) method used by researchers for example to analyze the content of Al (Mirza et al., 2017; Mold et al., 2018) has also become popular.

### Sample Preparation

Solid samples such as human brain parts have to be mineralized before any test, which involves getting rid of the organic matrix, decomposition of the sparingly soluble compounds, and transferring the components without loss into the solution. The literature review shows that microwave mineralization using HNO or mixtures of HNO and H O is used in most cases (see examples in Table 2).

**Table 2:** Sample conditions of microwave mineralization of human brain samples.

**Sample weight**	**Substances**	**The volume to made up**	**References**
0.3 g wet weight was dried to a constant weight at 37C	Mixture of 1 ml 15.8 M HNO_3_ and 1 ml 30% w/v H_2_O_2_	5 ml ultrapure water	Mold et al., 2018
100–500 mg dried samples	Mixture of 2.5 ml of HNO_3_≥65% and 1.0 ml of H_2_O_2_≥30%	50 ml ultrapure water	Becker et al., 2007b
25 mg of dried samples	2.0 ml 70% HNO_3_ and 0.5 ml 30% H_2_O_2_	Brak danych	Popescu et al., 2009c
0.1 mg of freeze-dried sample	HNO_3_	Brak danych	Warren et al., 1960

## Nuclear Methods

### XRF

XRF fluorescence spectrometry can also be used to determine the content of elements. It works well for analysis of ingredients found in both large and small quantities, which distinguishes this method from the other ones usually used in instrumental analysis.

The XRF method is based on the induction of the characteristic X-ray radiation by means of the radiation coming from an X-ray tube (with a rhodium or copper) that emits a continuous radiation spectrum. This radiation is directed to the test sample (formed in the form of a compressed tablet or molten with oxoborate of lithium beads) through a beryllium window and a system of brass and aluminum filters. The characteristic x-ray radiation that arises after reflection from the sample passes through a collimator, which concentrates the beam on the analyzing crystal. Bent at a certain angle, specific for a given element, the characteristic radiation beam is analyzed by a flow or fluorescent detector. On this basis, the computer system determines the content of the analyzed element.

The analysis of elements with an atomic number <6 is not very effective due to the extremely high ionization energy of internal atomic shells.

The biggest advantages of this method are: the possibility of analyzing many elements at the same time, short duration of the analysis, easy sample preparation and the fact that the sample is not destroyed during the analysis. The limitations may be, in turn, expensive equipment, lack of information on oxidation levels of elements, and fairly high limits of quantification. This technique, connected with microscopy (James et al., 2011) allows to investigate the distribution of elements inside the tissue.

X-ray fluorescence spectroscopy is an excellent method for studying elements at the ppm level (to parts per million or more). This method appears to be a well-established technique for quantitative determination of numerous metals in small areas of tissue samples (11–13). If the sample is additionally exposed to an X-ray beam applying X-ray fluorescence microscopy (XFM), the method can be useful to map large brain slices. The important advantage of the XRF is the possibility to obtaining high-resolution maps visualizing spatial distribution (to sub- 100 nm) of a large number of elements in biological samples. There are different approaches to imaging subcellular details by XFM, such as e.g., electron-probe X-ray microanalysis or EPXMA, etc. Until several years ago, XFM was not widely available to bio-medical communities and rarely offered resolution better than several microns. This has changed drastically with the development of third-generation synchrotrons (USA [APS], France [ESRF], Japan [SPring8]) offering relevant spatial map resolution able to perform quantitative elemental imaging of hydrated biological specimens with submicron-resolution. Moreover, SXRF microscopy is able to provide information concerning the oxidation state of an element and even coordination environment (micro-XANES spectroscopy; Shahata et al., 2015). Synchrotron X-ray fluorescence technique is described precisely in the following research and review papers (James et al., 2011; Majumdaz et al., 2012; Pushie et al., 2014; Niemiec et al., 2015; Takano et al., 2017). To map small areas, traditional point-to-point x-ray fluorescence imaging may be applied, but to map large areas, rapid-scanning x-ray fluorescence mapping (RS-XRF) employing appropriate software is able to substantially decrease the scanning time (Fahrni, 2007).

The XRF has many successful applications in diagnostics. Wandzilak et al. (2015) proved a statistically significant relationship between the concentration of selected elements such as P, S, Ca, and Fe and the severity of cancer. The authors showed that changes in the concentration of these elements are related to the tumor's grade of malignancy. The obtained results suggested that the examined transition metals appear to be important during carcinogenesis. Another example of a very useful application of the above method is the determination of P, S, K, Ca, Fe, Cu, Zn, and Se changes occurring within hippocampal formation as a result of high-fat and carbohydrate-restricted ketogenic diet (KD) (Snigireva and Snigirev, 2006). In other paper (Miller et al., 2006), a method for the imaging of the spatial distribution of chosen metals (Ca, Fe, Cu, and Zn) in the brain of a mouse model of Alzheimer's disease by synchrotron radiation (SR) based X-ray fluorescence analysis (XRF) was described. Owing to synchrotron X-ray fluorescence (SXRF) microprobe, metal ion such as iron (Fe), copper (Cu), and zinc (Zn) accumulation has been confirmed in brain tissue of Alzheimer's disease (AD) patients (Tiiman et al., 2013). Some studies describe rapid-scanning x-ray fluorescence (RS-XRF) to measuring iron content in the brain slices from Parkinson's disease (PD) and a number of neurodegenerative diseases (Kikuchi et al., 2004; Yang et al., 2005).

### Fluorescent Probes

Recently, an increase in the kind of fluorescent metal ion probes obtained by combining a fluorophore with a known metal ion chelator has been observed. Fluorescence probes are based on fluorescence quenching or switching mechanisms known as “turn-off probes” or “turn-on”, respectively. The “turn-on” probes appear to be more efficient for specific events, mainly because of increased sensitivity and reducing of the false positive signals. Fluorescent probes through absorbing light of a specific wavelength and emitting light of usually longer wavelength can be used as a marker for microscopical analysis. This method has been used for the real time imaging of small molecules in living cells (Chen et al., 2013; Takano et al., 2017). It turned out to be efficient for targeting sulfane sulfur, which was presented in the study performed by Gao et al. (2018). Thanks to the fact that their probes had deep tissue penetration and minimal interference from background autofluorescence as well as mitochondria-targeting properties, they were able to provide an *in vivo* imaging of sulfane sulfur in living cells. This also allowed detecting the level changes of sulfane sulfur.

Nandre et al. have shown an efficient “turn-on” fluorescent probe BTP-1 based on the benzo-thiazolo-pyrimidine to selectively measure and monitor Fe^3+^ alterations in living cells. It turned out to have excellent selectivity and a low detection limit as well as low cost and easy preparation(Takano et al., 2017; Gao et al., 2018).

Another “turn on” fluorescent probe BOD-NHOH based on hydroxylamine oxidation has been proposed by Wang et al. (Nandre et al., 2014) for assessing intracellular ferric ion levels.

### NAA

The method uses the phenomenon of converting stable nuclear nuclei into radioactive ones and measuring the characteristic radiation emitted by these nuclei. The advantage of the method is that it is non-destructive, provides high sensitivity and the ability to determine 50–65 elements at the same time, has low detection limit, and requires no preliminary stage of sample preparation. One of the most important advantages is that most sample matrices appear to be “transparent” during activation. This is because the main elements constituting the sample matrix (hydrogen, carbon, oxygen, nitrogen, phosphorus, and silicon) do not form radioactive isotopes. This property makes NAA a method characterized by high sensitivity in the determination of trace elements—when the matrix elements seem to be absent, there is no basis for interference. The disadvantage of the method is that it is labor-intensive and time-consuming. All radioactive isotopes have different half-life times and can be divided into three categories: nuclides with a short time after the fissile breakdown (time may be less than a second and last up to several hours), nuclides with an average time after a half-life decay (the time may last from about 10 h to several days), nuclides with a long half-life (from several days to several weeks or even months). In addition, the NAA method provides information on the total concentration of elements, without distinguishing their chemical form and/or physical state; some elements are not possible to be determined, e.g., Pb; as access to the nuclear reactor is required in their case.

NAA was applied to assess chosen element concentrations in brain tissues of normal and demented individuals by Leite et al. (Wang et al., 2012). Concentrations of the following elements Br, Fe, K, Na, Rb, Se, and Zn were determined in the hippocampus and frontal cortex tissues. The above research confirmed that NAA is a useful method for human brain analysis. The study proved that high concentrations of Fe and Zn in the hippocampus could be responsible for neurodegenerative diseases. Another work used neutron activation analysis (NAA) for the determination of Na, K, Rb, and Cs in brain samples of AD patients (Bélavári et al., 2005). The authors compared NAA method with rapid spectrochemical ones such as ICP-AES and ICP-MS. They noticed a good agreement between the applied techniques for Na, K, and Rb, whereas cesium levels exhibited higher differences. The distribution of Na, K, Rb, and Cs in human brains was carried out by instrumental neutron activation analysis by Bélavári et al. (2004). The authors observed non-homogeneous distribution for sodium whereas for K, Rb, and Cs uniform distribution was proved. The authors measured the following concentrations: 7,440 μg Na g^−1^ dry weight, 12,800 μg K g^−1^, 14 μg Rb g^−1^, and 50 ng Cs g^−1^. Furthermore, they found a strong statistical significance between Rb and Cs content in brain tissue.

### PIXE

This method is based on the use of the so-called braking radiation. The sample subjected to ion bombardment (for this purpose protons produced in the accelerator with the energy of several MeV are most often used) begins to emit radiation in the X-ray field characteristic of the constituent elements. The intensity of this radiation is a measure of the content of individual analytes in the sample. Radiation arises from the elimination of electrons from the internal electron shells of the atoms that make up the sample. The electrons from the higher shells, therefore, of higher energy, complement the gaps of the lost electrons taking their place and emitting excess energy in the form of characteristic X-ray radiation.

### INAA

The neutron activation method is characterized by a particularly high precision as well as low limit of detection and determination. For these reasons, it is often used in the preparation of analytical standards. It involves bombardment of the tested sample with a neutron beam. They are produced most often in special generators and have an initial energy of ~14 MeV. As a result of braking processes on light elements, their energy can be adapted to current analytical needs. Neutrons, due to non-oblate collisions with the nuclei of the sample, cause the formation of artificial radionuclides. The intensity of the characteristic nuclear radiation of the resulting nuclides is measured and compared with the radioactivity of the applied standard. On this basis, the content of individual elements in the sample can be determined.

There are literature reports on research on the determination of elements in human brain samples carried out for comparison between nuclear and spectroscopic methods. Andrási et al. (1999) described in their work the content of Cu, Fe, Mn, Zn, Cd, Pb by ICP-AES, GFAAS, and INAA. The obtained results allowed to state that these methods are adequate for determining the above- mentioned elements. The only exception was the analysis of Cd and Pb content using the INAA method. In this case restrictions on the limit of determination have appeared.

In the study (Császma et al., 2003) the performance evaluation of several methods for the determination of Mo and Mn content was carried out. The content of molybdenum was investigated using the ETAAS and ICP-MS methods, while the manganese content was additionally tested using ICP-AES and NAA. The above methods were compared in terms of accuracy, precision, detection limit, time of analysis, and the required amount of sample. The obtained results showed that both the ETAAS and ICP-MS method is suitable for Mo content analysis at the level of ng/ml, however, in the case of the ETAAS method it is necessary to concentrate the sample, which extends the time of analysis. In the case of Mn content, all the methods assessed (ETAAS, ICP-AES, ICP-MS, and NAA) proved adequate. For both analyzed elements, regardless of the method used, the results were obtained.

### Sample Preparation

On the contrary to the spectroscopis methods, samples used in nuclear analysis are not mineralized, as these methods require solid sample. The tissues are dried, sometimes oxidized before drying process by nitrous acid, and then simply grinded for peletting. To avoid decomposition of the tissue, lyophilization is sometimes preferred. The simplicity of sample preparation can be perceived as an advantage of these methods.

## Distribution of Trace Elements

The data presented in Table 3 show that for the quantitative and qualitative analysis of elements in human brain samples, the ICP-MS and ICP-AES methods are most often used. This is mainly due to the low limits of quantification of these methods, the ability to determine most elements of the periodic table and relatively short analysis time. Alkali metal contents are most often determined using the FAES, ICP-MS, NAA, and INAA methods. Activation analysis is also used for the determination of rare earth elements. In order to determine the distribution of individual elements, the SEM-EDS methods are used. Speciation analysis of elements found in the human brain can be performed using combined techniques such as HPLC-ICP-MS. To allow the readers have some reference values, the most important trace elements with analysis examples will be described below.

**Table 3:** Literature values (per dry weight) of various trace elements in different parts of human brain.

**Harrison et al., 1968**	**Region**	**Cu ug/g**	**Zn ug/g**	**Mg ug/g**	**Fe ug/g**								
Method: atomic absorption spectroscopy	Front gray	38 ± 10.6 (23–60)	95 ± 30.9 (53–228)	720 ± 104.1 (529–907)	242 ± 45.6 (131–343)								
	Front white	22 ± 11.8 (12–82)	45 ± 11.8 (24–79)	543 ± 64.1 (424–681)	185 ± 42.5 (107–253)								
	Hippocampus	29 ± 11.2 (13–70)	95 ± 37.7 (36–288)	658 ± 104.5 (303–942)	219 ± 36.9 (115–294)								
	Caudate nucleus	42 ± 15.9 (31–63)	81 ± 20.8 (53–172)	746 ± 96.5 (528–966)	535 ± 132.3 (243–763)								
	Thalamus	21 ± 4.3 (13–28)	58 ± 9.0 (45–70)	630 ± 54.8 (551–719)	217 ± 63.2 (144–314)								
**Greiner et al.**, **1975**	**Region**	**Ca ug/g**	**Cu ug/g**	**Mg ug/g**	**Zn ug/g**								
Method: atomic absorption spectrophotometry	White matter	103	9.03	137	7.01								
	Gray matter	79	7.07	112	9.09								
	Thalamus	115	7.02	155	10								
	Nucleus caudatus	71	7.01	132	11.5								
**Höck et al.**, **1975****; Burk et al.**, **2014**	**Region**	**Co ug/g**	**Fe ug/g**	**Rb ug/g**	**Se ug/g**	**Zn ug/g**							
	Gyrus postcentralis	3.6 ± 1.75	3.05 ± 0.22	1.15 ± 0.11	7.1 ± 1.02	5.92 ± 0.46							
	Hippocampus	10.28 ± 1.63	2.38 ± 0.19	2.11 ± 0.33	8.57 ± 0.96	9.63 ± 0.47							
	Nucleus caudatus	6.07 ± 0.99	8.3 ± 0.46	2.27 ± 0.13	8.6 ± 0.83	8.63 ± 0.36							
	Anterior thalamus	5.1 ± 0.82	3.29 ± 0.32	1.82 ± 0.24	9.02 ± 1.19	5.98 ± 0.58							
	Insula	4.44 ± 0.14	2.94 ± 0.1	1.97 ± 0.18	5.78 ± 1.65	9.60 ± 0.37							
**Duflou et al.**, **1989**	**Region**	**K ug/g**	**Ca ug/g**	**Mn ug/g**	**Fe ug/g**	**Cu ug/g**	**Zn ug/g**	**Se ug/g**	**Rb ug/g**				
Method: PIXE	Gurys cinguli	17,900 ± 1,900	510 ± 300	1.3 ± 0.12	199 ± 39	24.9 ± 5.9	74.3 ± 7.9	0.9 ± 0.19	17 ± 2.6				
	Gyrus postcentralis	16,400 ± 2,200	357 ± 60	1.38 ± 0.21	277 ± 74	25.5 ± 5.1	63.1 ± 4.5	0.87 ± 0.1	14.8 ± 2.9				
	Uncus hippocampi	17,000 ± 2,400	307 ± 39	1.54 ± 0.29	225 ± 54	22.0 ± 6.2	85 ± 19	0.83 ± 0.14	16.9 ± 3.1				
	Nucleus caudatus	16,800 ± 1,000	321 ± 40	2.48 ± 0.26	520 ± 180	31.3 ± 3.8	73.3 ± 4.0	1.03 ± 0.16	18.4 ± 2.9				
	Anterior thalamus	15,000 ± 2,100	380 ± 140	1.84 ± 0.26	306 ± 74	21.2 ± 7.6	56.5 ± 5.8	0.87 ± 0.19	16.4 ± 3.4				
**Rajan et al.**, **1997**	**Region**	**Cu ug/g**	**Al ug/g**	**Si ug/g**	**As ug/g**	**Ni ug/g**	**Pb ug/g**	**Cd ug/g**	**Cr ug/g**	**Zn ug/g**	**Fe ug/g**	**Ca ug/g**	**Mg ug/g**
Method: lCPAES	Frontal cerebrum	4 ± 1.7 (2–6.4)	113 ± 54 (26–181)	25.5 ± 15.7 (7–51.7)	0.84 ± 0.35 (0.47–1.69)	0.26 ± 0.02 (0.24–0.31)	0.42 ± 0,28 (0.2–1.14)	0.022 ± 0.016 (0.007–0.056)	0.15 ± 0.05 (0.05–0.22)	7.5 ± 0.5 (7–8)	50.8 ± 13.6 (33–71.6)	178 ± 64 (98–251)	158 ± 29 (121–200)
	Postcentral gyrus	4.3 ± 2.1 (1.7–4.9)	69 ± 34 (21–128)	17.4 ± 6.8 (8–25.4)	0.67 ± 0.33 (0.2–1.36)	0.26 ± 0.02 (0.25–0.30)	0.40 ± 0.13 (0.29–0.66)	0.021 ± 0.01 (0.007–0.036)	0.13 ± 0.07 (0.04–0.22)	9 ± 0.5 (9–10)	38.7 ± 15.5 (14.2–64.3)	178 ± 100 (59–391)	124 ± 35 (64–182)
	Hippocampus	4.0 ± 1.0 (2–5.1)	112 ± 56 (47–221)	28 ± 14.2 (19–87.4)	1.1 ± 0.7 (0.2–1.74)	0.27 ± 0.01 (0.26–0.30)	0.9 ± 0.7 (0.15–2.23)	0.022 ± 0.005 (0.016–0.032)	0.1 ± 0.08 (0.01–0.29)	6.5 ± 0.5	26.6 ± 7.6	125 ± 51 (54–186)	116 ± 17 (84–131)
	Caudate nucleus	N/A	N/A	N/A	N/A	N/A	N/A	N/A	N/A	N/A	N/A	N/A	N/A
	Thalamus	3.5 ± 1.4 (1.0–4.7)	129 ± 76 (31–241)	28 ± 14.2 (11–52.9)	0.98 ± 0.55 (0.11–1.86)	0.19 ± 0.11 (0.08–0.31)	0.71 ± 0.42 (0.48–1.64)	0.05 ± 0.04 (0.02–0.034)	0.14 ± 0.09 (0.06–0.14)	7.5 ± 0.5	41.8 ±18.5	158 ± 72	113 ± 30
**Krebs et al.**, **2014**	**Region**	**Cu um/g**	**Zn ug/g**	**Mn ug/g**	**Fe ug/g**	**Mg ug/g**	**Ca ug/g**						
Method: coupled plasma mass spectrometry	Frontal cortex	3.94 ± 0.99 (2.28–6.28)	12.2 ± 3.48 (8.2–20.9)	0.149 ± 0.022 (0.11–0.21)	27.4 ± 8.7 (16.6–55.1)	53.1 ± 6.3 (36–60.4)	153.9 ± 47.5 (75.9–270.4)						
	Front white	4.42 ± 1.22 (2.14–7.36)	10.7 ± 2.14 (6.3–20.7)	0.325 ± 0085 (0.18–0.46)	46.7 ± 11.7 (28–83.9)	123.7 ± 24.7 (94.1–163.6)	61.2 ± 8.7 (46.5–85)						
	Thalamus	4.83 ± 1.53 (2.33–7.61)	12.3 ± 3.23 (9–29.5)	0.315 ± 0.076 (0.2–0.44)	49 ± 10.9 (34.5–80.9)	99.1 ± 20.5 (57.4–134.1)	66.6 ± 10.1 (50–87)						
	Caudate nucleus	5.6 ± 1.25 (4.17–10.03)	12.4 ± 2.9 (8.5–19.1)	0.383 ± 0.075 (0.26–0.47)	106 ± 26 (61.4–171)	94.9 ± 18.6 (73.2–124.1)	72.6 ± 13.2 (56.5–106.5)						

### Iron

Iron is absorbed in the intestine through ferrireductase activity in the luminal side and the divalent metal transporter 1 on the apical membrane of the enterocytes (Gunshin et al., 1997) and is regulated depending on the iron levels.

Being a cofactor in the synthesis of myelin as well as neurotransmitters, and, because of its role in oxidative metabolism (inducer of reactive oxygen species), iron plays an important role in proper brain functioning. It is involved in oxygen transport, glucose metabolism, electron transport, synthesis of myelin, neurotransmitters, and DNA replication. Unfortunately, excessive accumulation of iron may lead to a formation of highly reactive hydroxyl radicals.

Iron transportation to the brain tissue relies on the previously mentioned blood-brain barrier and blood-CSF barrier. Plasmatic iron access is limited by the BBB, so ions are transported to the brain by plasma transferrin through an interaction between circulatory transferrin and transferrin receptors (TfR) (Burk et al., 2014) in capillars because of a high density of TfR in capillary endothelial cells (Connor, 1994). After releasing iron in the endothelial cells, apo-transferrin is released into the circulation.

Within the brain tissue, iron can be divided into heme iron and non-heme iron and it was revealed for the first time by the use of histochemical analysis (Prussian blue or Perls' stain). Heme iron can be found in hemoglobin, non-heme iron is present in metalloproteins, low molecular weight complexes, storage proteins and ionic iron. However, there is no possibility to count heme and non-heme fraction of the iron after a mineralization when performing analysis by any aforementioned methods.

Iron can be found mostly within the brain regions responsible for motor functions, where two to three times more of Fe has been found (Zecca et al., 2004). Popescu et al. (2009a) report that gray matter structures contain more iron than white matter structures. The highest iron concentration was found within globus pallidus, substantia nigra, putamen, caudate nucleus, red nucleus, dentate nucleus, and locus coeruleus, which can indicate the vulnerability of these structures to effects of iron levels' disturbance in movement disorders (Dexter et al., 1989; Haacke et al., 2005; Popescu et al., 2009b).

Stüber et al. (2014) performed an iron mapping study using MRI. Within the motor/somatosensory cortex, iron distribution was found to have a laminar structure in gray matter, overlapping the myelinated bands of Baillarger. Moreover, a narrow iron-rich band in the white matter was present, close to the border with the cortex, and uneven distribution in other white matter regions. Visual cortex also appeared to have highly concentreted iron regions, mainly in striatum.

### Copper

Copper is obtained orally from the daily diet and is excreted via the biliary system. It is placed in the brain from peripheral copper through blood-brain barrier and/or the blood-cerebrospinal fluid barrier. Copper is transported into the brain parenchyma through the BBB mostly as a free ion, where it is utilized and released into the CSF. Choroid epithelial cells absorb copper from the CSF, and thus copper homeostasis is determined (Zheng and Monnot, 2012). As well as iron, copper is a component/cofactor of various enzymes, which have a crucial role in biological reactions such as antioxidation, energy metabolism, iron metabolism, neuropeptide (peptidylglycine-α-amidating enzyme), and neurotransmitter (dopamine-β-monoxygenase) synthesis (Scheiber and Dringen, 2013).

High copper levels were discovered in the substantia nigra, lous coeruleus (both containing catecholaminergic cells) (Davies et al., 2013), dentate nucleus, basal ganglia, hippocampus, and cerebellum (Warren et al., 1960; Becker et al., 2007b, Popescu et al., 2009a,c).

Gray matter was found to have higher copper concentration than white matter (Dobrowolska et al., 2008), however copper levels in the thalamus were found to be lower than in any other gray matter regions (Smeyers-Verbeke et al., 1974). Becker and Salver claim that glial cells have higher copper levels than neurons (Becker and Salber, 2010), mostly within the ventricular region of the brain (Szerdahelyi and Kása, 1986).

### Zinc

Zinc is an extremely important element, which is required by nearly 300 enzymes for their proper action. Zinc exits in the brain tissue mostly as a component of metaloproteins (90%) (Frederickson, 1989) and in the presynaptic vesicles (Howell and Frederickson, 1990) (it plays a role in synaptic neurotransmission and serves as an endogenous neuromodulator of various receptors).

Serum contains three different forms of zinc: the low molecular weight ligand-bound form, free Zn2+ ion, and the protein-bound form (mostly to albumin), which is the largest component of serum zinc.

Zinc transportation into the brain relies on BBB and the blood-CSF barrier. Because of incomplete development of BBB in the early postnatal period, protein-bound, and non-protein-bound zinc is able to freely pass through the BBB. There are four putative zinc transporters (ZnT-1–ZnT-4), which are suspected of zinc transportation, especially ZnT-1, associated with zinc efflux (Tsuda, 1997). 65Zn- histidine complex is suspected of being more stable in CSF than in serum and is linked with high Zn uptake in the brain parenchymal cells.

Zinc stabilizes the myelin structure, and therefore is highly concentrated within the white matter (Popescu et al., 2009a). High levels of this metal were also found in the hippocampus (in the hilus region and lucidum layer) and amygdalia (especially the amygdalopiriform transition and the amygdalohippocampal transition areas), which are rich in zincergic neurons (Mocchegiani et al., 2005) as well as in the dentate gyrus.

### Selenium

It is a very important element taking part, in various brain functions such as motoric performance, coordination, memory, and cognition, acting also as a neurotransmitter. Unlike other trace metals, selenium exists as a component of the amino acid selenocysteine. It has protective properties against oxidative damage (Burk et al., 2014), therefore selenium deficiency may cause irreversible changes in neuronal cells.

Brain tissue was found to be poor in selenium. Gray matter has higher concentrations of Se than white matter, as studies report (Caito et al., 2011). Ramos et al. (2015) in their study reported high selenium levels in putamen and the parietal inferior lobule. In different studies caudate nucleus, putamen, globus pallidus (Ejima et al., 1996), posterior occipital lobe, cerebellar vermix (Höck et al., 1975) were mentioned. Lower Se levels were reported in the hippocampus, amygdalia, as well as medulla and the cerebellum (Ramos et al., 2015).

### Manganese

This is an element that allows the functioning of many different enzyme families such as transferases, isomerases, ligases, hydrolases, transferases, and oxidoreductases. Among many different functions, Mn is involved in the regulation of blood sugar, the production of cell energy, reproduction, digestion, bone growth, blood clotting, immune function, amino acid, lipid, protein, and carbohydrate metabolism, protein glycosylation and the detoxification of superoxide free radicals (Markesbery et al., 1984; Aschner and Aschner, 2005; Roth, 2006). It is absorbed from the intestine and eliminated into the bile.

Excessive manganese intake was proven to cause Parkinson's disease and dementia. Mn is mostly concentrated in the globus pallidus, hypothalamus, nucleus caudatus, corpus pineale, and putamen (Martinez-Finley et al., 2013). Moreover, gray matter structures within the cerebellum were found to contain higher levels of Mn than gray matter structures in the cerebrum. On the other hand, low levels of Mn were found in the genu of corpus callosum, cerebral peduncule, corticospinal tract, pyramid, and corpus medullare of the cerebellum. Pallidal index was found to be an efficient biomarker to diagnose the early neurotoxic effects of Mn (Aschner et al., 2005).

### Cadmium

There are two ways of adsorbing cadmium into the brain: through the olfactory pathway, nasal mucosa or by disturbing the permeability of BBB (Li et al., 2014). Moreover, it is capable of passing to the fetus via the placenta and was detected in breast milk during lactation (López et al., 2006). Cd affects the brain tissue by damaging the DNA, peroxidation of lipids (Li et al., 2014), altering calcium homeostasis, and disturbing the functioning of different neurotransmitters (Korpela et al., 1986; Liu et al., 2008).

According to the study performed by Rajan et al. (1997), the highest levels of Cd were found in the thalamus, the cerebellum and the hippocampus. It was found in the choroid plexus at high concentrations, almost 2–3 times higher than in the brain cortex (Manton and Cook, 1984).

### Lead

Lead is a group IVa element, especially harmful to the brain tissue, with no known function in the human body. Pb affects cell signaling (through changes in the cell redox status, affecting second messengers interacting with protein components of the signaling cascade), cell redox status (through affecting reactive oxygen species' and reactive nitrogen species' production) and neurotransmission (disturbed function of acetylocholinoesterase, monoamino oxidase, tyrosine hydroxylase as well as decreased norepinephrine, epinephrine and dopamine levels in the hippocampus, cerebellum, and cerebral cortex). According to the study performed by Rajan et al. (1997), the highest levels of Pb were found in the hypothalamus.

## Conclusions

It seems that a post mortem analysis of the human brain can significantly settle its place in analytical chemistry because of its wider and wider use for understanding of many diseases. As the brain is not an easy matrix for such a procedure and there are many analytical methods for trace elements determination, the current review can be a starting point for choosing an appropriate method, dealing with common difficulties, and knowing what amount of various trace elements can be found in the analyzed samples.

Control of trace elements, especially of their spatial distributions is crucial as to fully elucidate their biochemical relevance. Many analytical methods for quantitative mapping of trace elements in cellular biology can be applied as a useful tool to explore the intracellular metal ion distribution accompanying the development of different diseases. Nowadays, spectrophotometry, despite such factors as low costs of the instruments or easy handling, remains a common technique only in laboratories of the developed countries. The progress in the field of colorimetric determination of metal ions in samples of biological origin is, undoubtedly, a new proposition and utilizations of chemical sensors. They are self-contained devices that can provide a measurable physical signal correlated with the chemical composition of the surrounding environment. Recently, the Zr-based metalorganic framework (UiO-66) or mesoporous TiO_2_ as solid chemical carriers for dithizone (Dz) have been described for sensitive and selective discrimination of trace levels of some toxic metal ions such as Cu(II), Pb(II), Hg(II), and Cd(II) at 10^−9^mol/dm^3^ in post-mortem biological samples (Shahat et al., 2013; Shahata et al., 2015).

However, at present, some more sophisticated analytical techniques with appropriate sensitivity are available for the evaluation and speciation of elements at trace levels in biological. We can enumerate inductively coupled plasma mass spectrometry (ICP-MS), secondary ion mass spectrometry (SIMS), atomic emission spectroscopy (AE), which can be applied in order to obtain precise measurements of metals, even at low concentrations. These methods, however, require isolation and purification of the cellular structures of interest to estimate metal distribution and speciation. This step of analysis is often associated with a disadvantageous process involving contamination of the sample by artifacts. Moreover, these methods are not characterized by sufficient spatial sensitivity and they completely destroy the analyzed tissue.

Therefore, microscopy imaging techniques which are non-destructive, appear to be better suited to examine the subcellular distribution of metal ions. Although the use of XFM or synchrotron-based X-ray fluorescence microscopy (SXRF, SRIXE, or microXRF) in biomedical studies of tissues of even single cells has become common in recent years, sample preparation still remains unclear and could be a source of artifacts (James et al., 2011). It should be emphasized, however, that accurate determination of elements utilizing the recorded spectra requires appropriate calibration and relevant operating conditions. Moreover, by the use of membrane diffusible fluorescent probes, the thermodynamic and kinetic availability of metal ions (Kikuchi et al., 2004; Yang et al., 2005) is possible to evaluate. The applications of X-ray fluorescence microprobe imaging in biology and medicine is a theme of interesting review papers (Paunesku et al., 2006).

## Author Contributions

All authors listed have made a substantial, direct and intellectual contribution to the work, and approved it for publication.

### Conflict of Interest Statement

The authors declare that the research was conducted in the absence of any commercial or financial relationships that could be construed as a potential conflict of interest.
